# A CT-Based Radiomics Nomogram Model for Differentiating Primary Malignant Melanoma of the Esophagus from Esophageal Squamous Cell Carcinoma

**DOI:** 10.1155/2023/6057196

**Published:** 2023-02-20

**Authors:** Yan-Jie Shi, Hai-Tao Zhu, Shuo Yan, Xiao-Ting Li, Xiao-Yan Zhang, Ying-Shi Sun

**Affiliations:** Key Laboratory of Carcinogenesis and Translational Research (Ministry of Education), Department of Radiology, Peking University Cancer Hospital & Institute, No. 52 Fu Cheng Road, Hai Dian District, Beijing 100142, China

## Abstract

**Objective:**

The diagnosis of primary malignant melanoma of the esophagus (PMME) before treatment is essential for clinical decision-making. However, PMME may be misdiagnosed as esophageal squamous cell carcinoma (ESCC) sometimes. This research is aimed at devising a radiomics nomogram model of CT for distinguishing PMME from ESCC.

**Methods:**

In this retrospective analysis, 122 individuals with proven pathologically PMME (*n* = 28) and ESCC (*n* = 94) were registered from our hospital. PyRadiomics was applied to derive radiomics features from plain and enhanced CT images after resampling image into an isotropic resolution of 0.625 × 0.625 × 0.625 mm^3^. The diagnostic efficiency of the model was evaluated by an independent validation group.

**Results:**

For the purpose of differentiation between PMME and ESCC, a radiomics model was constructed using 5 radiomics features obtained from nonenhanced CT and 4 radiomics features derived from enhanced CT. A radiomics model including multiple radiomics features showed excellent discrimination efficiency with AUCs of 0.975 and 0.906 in the primary and validation cohorts, respectively. Then, a radiomics nomogram model was developed. The decision curve analysis has shown remarkable performance of this nomogram model for distinguishing PMME from ESCC.

**Conclusions:**

The proposed radiomics nomogram model based on CT could be used for distinguishing PMME from ESCC. Moreover, this model also contributed to helping clinicians determine an appropriate treatment strategy for esophageal neoplasms.

## 1. Introduction

Malignant tumors that develop from melanocytes located in epithelial basal layer of esophagus are known as PMME [1]. The PMME is an odd disease that accounts for approximately 0.1-0.2% of all esophageal malignant neoplasms, 0.5% of all non-cutaneous melanomas, and 5.6% of all gastrointestinal tract melanomas [2–4]. Furthermore, it is a deadly neoplasm with just a 4-5% five-year survival rate [1, 5]. The aggressive excision of the tumor is still the major treatment for PMME. Patients with PMME have an 82.28% surgical resection rate, but the vast majority of them still die from the disease disseminating after the surgery [6]. There are just 4.5 months between primary surgery and recurrence, and the chance of recurrence is quite high, with a shorter recurrence free survival of just 2 months in patients who received only surgery without adjuvant therapy [7]. Patients with PMME may have a better prognosis if a greater number of lymph nodes are removed during surgery [8]. Despite the availability of endoscopic biopsy, the preoperative pathologic diagnosis of PMME is low, and 20-50% of PMME are reported to be misdiagnosed as an esophageal carcinoma owing to the inadequacy of melanin granules in PMME [8, 9]. Chemotherapy and chemoradiotherapy have not been shown to be effective for PMME, in contrast to ESCC, and immune-checkpoint inhibitors represent a novel treatment strategy for PMME [9]. Therefore, a greater understanding of the features of PMME and its distinction from ESCC will increase the efficacy of the diagnosis and the selection of a suitable treatment plan.

The chest CT remains routinely applied for diagnosing, restaging, and monitoring treatment therapy for esophageal neoplasms. The CT imaging features of PMME have been reviewed, and the qualitative and quantitative CT analyses have excellent performance with an AUC of 0.969 to differentiate PMME from ESCC [10]. However, the CT imaging information for distinguishing PMME from ESCC is assessed subjectively. Recently, radiomics, as an emerging tool, derives lots of features directly from images and convert images into quantitatively mineable data [11]. Several studies have shown substantial diagnostic value in esophageal tumor detection, tumor and nodal staging, predicting prognosis, and assessing treatment response. These radiomics models have exhibited higher performance in evaluating esophageal neoplasms and predicting prognosis [11–15]. However, no prior radiomics investigations have been undertaken to differentiate PMME from ESCC.

Herein, we hypothesize that a radiomics model based on CT may provide more optimal identification characteristics of esophageal neoplasms and have potential value in preoperative differentiation between PMME and ESCC. Given the exceedingly unusual prevalence of PMME, there are few investigations that include large numbers of patients. Consequently, we have employed a relatively large sample size in this work to develop and validate a CT radiomics model that can differentiate PMME from ESCC.

## 2. Materials and Methods

### 2.1. Ethical Statement

The Peking University Cancer Hospital ethics committee had given their approval for this retrospective investigation. The informed consent requirement was waived.

### 2.2. Patients

**Figure 1:**
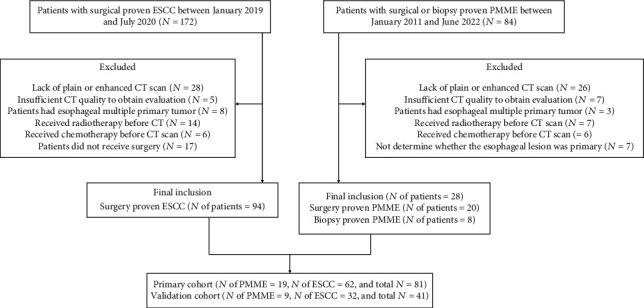
Representation of patient flowchart.

28 patients with surgically or biopsy-pathologically confirmed PMME were enrolled in our hospital from January 2011 to June 2022. 94 patients with surgically pathologically confirmed ESCC were identified at our hospital between January 2019 and July 2020. The following conditions were used as inclusion criteria: (1) patients underwent plain and enhanced chest CT examinations before treatment; (2) PMME was pathologically confirmed by surgery or biopsy, and ESCC was pathologically confirmed by surgery; (3) the diagnostic quality of the images was adequate for analyzing lesions; and (4) sex, age, and clinical characteristics were accessible. The following were the criteria for exclusion: (1) patients who had prior esophageal neoplasm treatment before their initial chest CT scan, (2) patients whose tumor was not observed at the CT scan, (3) patients who presented with multiple primary malignancies, (4) patients with poor quality CT images, (5) not determining whether the esophageal lesion was primary. In conclusion, 122 patients were included in this investigation. The individuals were randomly assigned to the primary and validation cohorts in a 2 : 1 ratio.  Figure 1 shows the whole patient enrollment procedure.

Two radiologists determined the location of the tumor using chest CT in accordance with the 8th edition of the American Joint Committee on Cancer (AJCC) staging [16]. Each patient's age, gender, and histopathological information were recorded according to the clinical and pathological reports.

### 2.3. CT Protocol

All patients fasted for 2-4 hours before the CT scan. Patients were not administered an oral contrast agent. Standard plain and contrast-enhanced CT scanning was performed on each patient applying the Discovery CT750 HD scanner. All patients had plain and contrast-enhanced CT scans during a single breath hold while in the supine position. The normal starting point for the scan was 2.0 cm above the lung apices, and it continued into the adrenal glands. We employed the following imaging protocol: a helical pitch of 0.9, a gantry rotation speed of 0.6 s, a tube voltage of 120 kVp, an autoregulation of mA (200-400 mA), a detector collimation of 64 × 1.25 mm, and a noise index of 9. Employing the Advantage Workstation 4.4, a width of 5.0 mm was used to reconstruct the axial, coronal, and sagittal reformations. Iohexol, a nonionic contrast medium, was administered at a rate of 3.0 mL/s into the median cubital vein at a dosage of 1.5 mL/kg. The delayed time of enhanced CT scan was 55 seconds after injecting the contrast medium.

### 2.4. Tumor Segmentation

In this investigation, tumor delineation and feature extraction were performed using plain and enhanced axial CT scans. The pretreatment CT was analyzed by a radiologist (Dr. Shi, with 12 years of expertise in gastrointestinal neoplasms). The radiologist manually outlined the regions of interests (ROIs) including the entire tumor along the tumor boundary using ITK-SNAP software. Necrosis and air areas were excluded from the ROIs. Another radiologist (Dr. Yan, who has 6 years of experience with gastrointestinal tumors) drew the ROIs of tumors in about 40 randomly selected samples from the primary group to evaluate the reproducibility of the extracted features. The repeatability of two radiologists was analyzed using the interclass correlation coefficient (ICC) indicator. Generally, extracted characteristics with an ICC greater than 0.75 were considered to have high repeatability agreement.

### 2.5. Extraction of Radiomics Features and Model Development

Radiomics features were extracted by PyRadiomics [17] after resampling the image into an isotropic resolution of 0.625 × 0.625 × 0.625 mm^3^. For plain images and contrast-enhanced images, 851 features, comprising 107 original features (14 shape features, 18 first-order features, and 75 texture features) and 744 wavelet-transformed features, were extracted from the ROIs [17].

By combining the features from plain images and contrast-enhanced images, a total of 1702 features were analyzed. The *T*-test and cross-correlation analysis were used to select features. The *T*-test was conducted in the training group to calculate the feature difference between ESCC and PMME. The characteristic was eliminated if the *P* value was greater than 0.05. The second step was to determine the correlation coefficient between each pair of remaining characteristics. The first step's characteristic was eliminated if the correlation coefficient's absolute value was more than 0.5. The reserved features were scaled to the range between 0 and 1 before classification. The SMOTE method was used to balance the sample sizes of ESCC and PMME in the primary group [18].

The radiomics model was developed from the aforementioned reserved quantitative features using Python and logistic regression with the least absolute shrinkage and selection operator (LASSO) in the training group. The LASSO applied a parameter *α* to control the weight of L1 regularization inside the loss function. ESCC was labeled as 0, and PMME was labeled as 1. It was determined using five-fold cross-validation that the best *α* in the training group is by maximizing the average area under the receiver operating characteristics curve (AUC). The best *α* was utilized to train all the subjects in the primary cohort for obtaining final radiomics model. Then, a nomogram based on radiomics model was established.

### 2.6. Statistical Analysis

We used SPSS 22.0 for Windows for statistical analysis (SPSS, Chicago, IL, United States). The Mann–Whitney *U*-test, or two-sample *t*-test, was used to compare the characteristics of patients. The radiomic features of PMME and ESCC were compared using the Student's *t*-test to see whether there were any statistically significant differences between them. In order to determine AUC, a receiver operating characteristic (ROC) curve analysis was carried out. The Maximum Youden index was utilized to identify the cutoff value to differentiate PMME from ESCC. In addition, sensitivity, specificity, positive prediction value (PPV), and negative prediction value (NPV) were calculated by maximizing Youden index (sensitivity + specificity − 1). All levels of statistical significance stated were two-sided. The *P* value < 0.05 was deemed statistically significant.

## 3. Results

### 3.1. Patient Characteristics

This study enrolled 28 patients with PMME (number men of 13 and women of 15; age from 35 to 79 years; mean age with 59.82 ± 11.32 years) and 94 patients with ESCC (number men of 80 and women of 14; age from 45 to 87 years; mean age with 63.39 ± 7.03 years). Tumor location (*P* = 0.367) and age (*P* = 0.123) did not vary significantly between the PMME and ESCC groups. There was a substantial gender difference in both the PMME and ESCC groups (*P* = 0.001). Comparison of the clinical characteristics of PMME and ESCC is compiled in Table S1. A comparison of the clinical characteristics of PMME and ESCC between the primary and validation cohorts is presented in Table 1. Age, gender, and pathological diagnosis showed no statistically significant differences between the primary and validation cohorts (all *P* > 0.05).

### 3.2. Radiomics Feature Extraction

Finally, for the differentiation of PMME and ESCC, we chose to build a model based on 5 radiomics characteristics derived from plain CT and 4 radiomics features derived from enhanced CT. The ICCs of these radiomics features were all larger than 0.75, further suggesting excellent agreement in reproducibility. The radiomics feature wavelet-HLL_ngtdm_Busyness (enhanced) had the highest AUC value of 0.707, followed by wavelet-HLL_glcm_Maximum Probability (plain) in the primary cohort.  Table 2 displays the comprehensive performance of these nine radiomics features.

### 3.3. Diagnostic Performance of Radiomics Model

The following formula was used to develop the radiomics model for differentiating PMME from ESCC:

(1) $$\begin{array}{c} \text{Radiomics signature}=0.13642422\times \text{original}\_\text{shape}\_\text{Sphericity}\left(\text{plain}\right)+0.21346447\times \text{wavelet}\_\text{HLH}\_\text{glrlm}\_\text{Low Gray Level Run Emphasis}\left(\text{plain}\right)-0.22369232\times \text{wavelet}\_\text{HLH}\_\text{glszm}\_\text{Size Zone Non Uniformity Normalized}\left(\text{plain}\right)-0.25650614\times \text{wavelet}\_\text{HLL}\_\text{glcm}\_\text{Maximum Probability}\left(\text{plain}\right)-0.25245768\times \text{wavelet}\_\text{LHH}\_\text{firstorder}\_\text{Minimum}\left(\text{plain}\right)-0.41765196\times \text{wavelet}\_\text{HLH}\_\text{firstorder}\_\text{Maximum}\left(\text{enhanced}\right)+0.27318268\times \text{wavelet}\_\text{HLL}\_\text{ngtdm}\_\text{Busyness}\left(\text{enhanced}\right)-0.21171556\times \text{wavelet}\_\text{LHH}\_\text{glcm}\_\text{Autocorrelation}\left(\text{enhanced}\right)+0.16697369\times \text{wavelet}\_\text{LHL}\_\text{glszm}\_\text{Gray Level Non Uniformity Normalized}\left(\text{enhanced}\right). \end{array}$$

**Figure 2:**
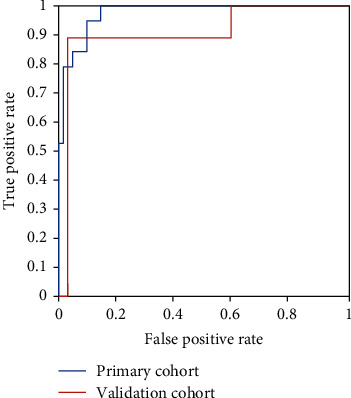
Diagnostic performance with area under curves (AUCs) of radiomics nomogram model. AUCs of radiomics nomogram model were 0.975 and 0.906 in primary and validation cohorts.

The optimal cutoff value of 0.50 was developed to identify the PMME and ESCC groups. Consequently, patients were split into two groups: those with PMME (radiomics signature > 0.50) and those with ESCC (radiomics signature ≤ 0.50). In the primary cohort, the radiomics model produced an AUC of 0.975 (95% confidence interval (CI): 0.948-1.000) and an accuracy of 91.4%, whereas in the validation cohort, the AUC was 0.906 (95% CI: 0.770-1.000) and the accuracy was 92.7%.  Table 3 and Figure 2 display the detailed efficiency of this radiomics model.

**Figure 3:**
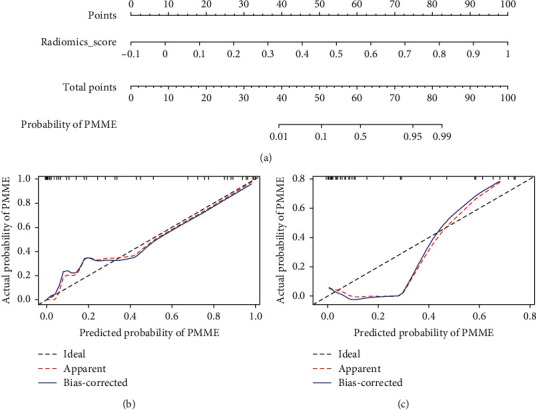
Radiomics nomogram model based on radiomics signature for differentiating PMME from ESCC. (a) The developed radiomics nomogram. (b) Calibration curves of radiomics nomogram model in primary cohort. (c) Calibration curves of radiomics nomogram model in validation cohort. The *y* axis showed the actual probability of PMME. The *x* axis showed the predicted probability of PMME. The black dotted line (ideal) presented a perfect prediction by an ideal model. The red dotted line (apparent) showed calibration curve of the nomogram model in this study. The blue line (bias-corrected) showed estimation of predicted values by bootstrapping. Calibration curves presented the calibration of radiomics nomogram model in terms of the agreement between the predicted probability of PMME and the actual outcomes of the PMME. The (b) and (c) suggested a better prediction of radiomics nomogram model for diagnosing PMME in primary and validation cohorts.

We established a nomogram based on the radiomics model.  Figure 3 illustrates the nomogram's corresponding calibration curves for the probability of PMME. The calibration curves demonstrated greater agreement and goodness-of-fit between the predicted PMME and the actual outcomes of the PMME.

### 3.4. Clinical Usefulness

**Figure 4:**
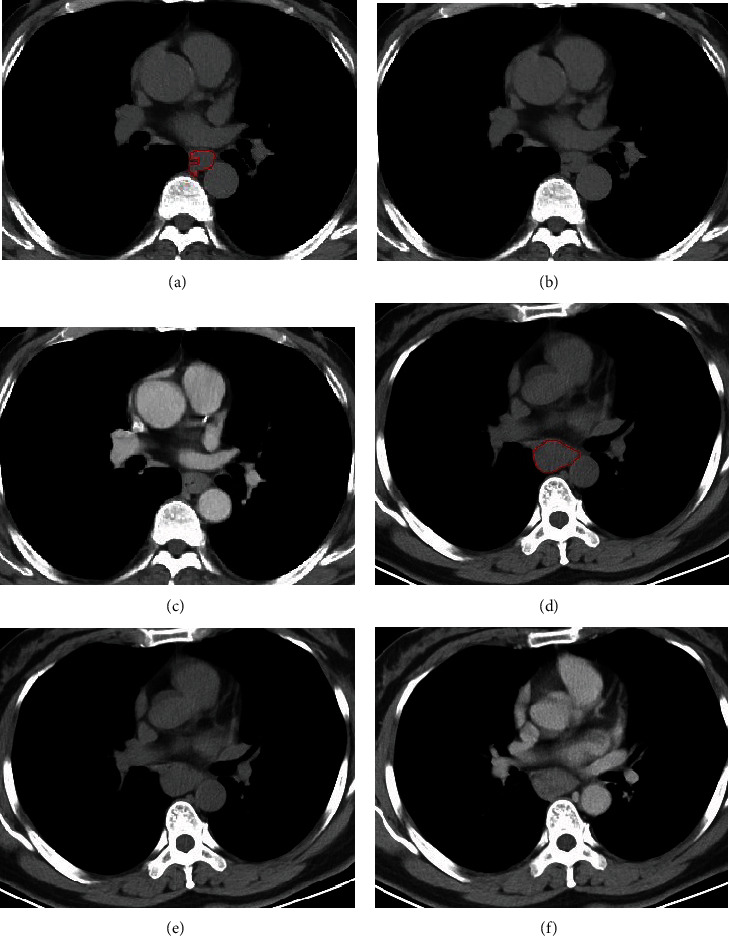
The cases of ROI segmentation and CT-based radiomics models for diagnosing ESCC and PMME. (a–c) 60-year-old man with ESCC. (a) The ROI was drawn along the tumor boundary in plain CT; (b) plain CT and (c) enhanced CT. Radiomics signature with score of 0.22 (less than the cutoff value of 0.50) suggested the diagnosis of ESCC. The radiomics nomogram model showed the radiomics score of 0.22 corresponded to 28 points, and the probability of diagnosing PMME was less than 0.01 indicating the diagnosis of ESCC. (d–f) 46-year-old women with PMME. (d) The ROI was drawn along the tumor boundary in plain CT; (e) plain CT and (f) enhanced CT. Radiomics signature with score of 0.98 (larger than the cutoff value of 0.50) suggested the diagnosis of PMME. The radiomics nomogram model showed the radiomics score of 0.98 corresponded to 98 points, and the probability of diagnosing PMME was larger than 0.99 stating the diagnosis of PMME.

The radiomics nomogram model, which is based on the radiomics signature, was created to provide clinicians with an easy method. The calibration curves of the nomogram demonstrated the model's exceptional performance for clinical applications. The probability of diagnosing PMME varied from 0% to 100%. A probability nearing 1 suggested a greater probability of PMME. This radiomics nomogram model might be advantageous for esophageal tumor patients (Figure 4).

## 4. Discussion

Survival rates after five years are just around 4% for those diagnosed with PMME, making it a very malignant tumor [6]. For individuals with PMME, early diagnosis and radical resection may be crucial for long-term survival [9]. For this reason, accurate and timely imaging differentiation of PMME from ESCC is essential because ESCC and PMME patients have drastically different clinical treatments and prognosis. PMME may present as dark pigmented masses, thereby suggesting the correct diagnosis. Unfortunately, pigmentation is not always visible upon visual examination, which may result in an incorrect diagnosis even in biopsy pathology [19]. In addition, a superficial biopsy may yield a negative result because PMME develops from melanocytes in the squamous epithelium's basal layer. CT is one of the most common noninvasive imaging modalities and plays a critical role in esophageal disease diagnosis [20]. Additionally, a bulky esophageal mass with neighboring mediastinal tissues compressed may be seen on a CT scan [21]. In comparison with ESCC, PMME always displays a clean boundary mass of esophagus and further depicting more blood supply with an obvious enhancement in the arterial phase [10]. Unlike ESCC, which has diffuse infiltration with an infiltrative border, PMME frequently has a wide polypoid appearance [10]. The differentiation of esophageal neoplasms may be aided by technological advancements in MRI, such as improved spatial and temporal resolution. Diffusion-weighted imaging and dynamic contrast enhancement are examples of more advanced imaging sequences that may be used to compute parameters about cellular density and blood perfusion [22]. High signal in T1WI and lower signal in T2WI are common MRI findings in melanin-rich melanoma. Many melanomas are atypical, and the signal of melanin is determined by the bleeding and melanin concentration of the tumor [23]. However, the radiological features of PMME in MRI have not been reported because of the rarity of PMME and the initial application of esophageal MRI in clinical practice. Another study is needed to investigate the radiological findings of PMME and evaluate whether the developed model based on MRI is suitable for clinical usefulness or not in the future. Endoscopy is a valuable diagnostic method for PMME. Endoscopy may reveal an elevated and pigmented tumor, frequently accompanied with ulcers [24]. Despite the fact that PMME is characterized by its black tone, it may also be purple, brown, or white [25]. Melanin spots around a protuberant tumor sometimes suggest intramural metastasis, indicating the diagnosis of PMME [24]. In the endoscopy, PMME can present as a cauliflower-like, protuberant, polypoid, or ulcerative mass [26].

Radiomics, which provide an objective method for assessing esophageal disease and can be used to discover correlations between radiomics properties and pathophysiology, may extract a large number of quantitative variables from digital images [27]. Recently, imaging-based radiomics analysis was applied in detection and classification of tumor, preoperative predicting histologic grade of tumor, and assessing treatment response to therapy in tumor [27–29]. Also, radiomics analysis has been successfully applied in estimating esophageal diseases for diagnosis, treatment response, and predicting prognosis. For patients with ESCC who had previously achieved pCR following nCRT and surgery, Qiu et al. reported that the radiomics nomogram model, including radiomics characteristics and clinical parameters, had an increased capacity to predict the postoperative recurrence risk [13]. Wu et al. discovered that a CT radiomics model may be utilized to predict LN metastases in ESCC patients prior to surgery [15]. What is more, using radiomics, Kao and Hsu discovered that it was possible to predict the full pathological response after neoadjuvant chemoradiotherapy in esophageal cancer [12]. The highest discriminative ability in OS prediction for ESCC patients with a C-index of 0.700 or above was shown to be attained by the integrating primary tumor and lymph node CT radiomics-clinical nomogram, according to research by Lu et al., and improved the calibration and classification for OS prediction compared with the clinical nomogram (C-index: 0.594-0.604) [14]. These previous studies showed that the radiomics signature outperformed other methods for evaluating esophageal diseases. So, we suspected that radiomics analysis based on CT imaging may be useful to differentiate PMEE and ESCC.

Due to PMME's very low prevalence, large-scale research with PMME patients is exceedingly uncommon. To create and verify a nomogram based on the CT radiomic signature, we employed a relatively large cohort in this work. We found that this nomogram showed excellent performance for distinguishing PMME from ESCC, with AUCs of 0.975 and 0.906 in the primary and validation cohorts, respectively. According to these results, the radiomics nomogram may be able to distinguish between PMME and ESCC, allowing clinicians to choose the most appropriate course of treatment. The utilization of plain and enhanced CT imaging contributed to the sturdiness and outstanding performance of this model. According to the research done by Shi et al., PMME is defined by a sheet of melanoma cells with rare stroma reactivity, but ESCC is characterized by extensive stroma around tumor nests. The expansive growth pattern of PMME was also different from that of ESCC, with an infiltrative pattern. PMME has a greater number of interstitial blood vessels for blood supply than ESCC. The 5 radiomics features extracted from plain CT may reflect the component of esophageal tumor. Additionally, the blood supply of an esophageal tumor may be reflected by four radiomic characteristics retrieved from enhanced CT. Consequently, the radiomic features may help to make the radiologic characteristics of esophageal neoplasms quantifiable and provide additional useful information.

Thus, we developed an easy method for differentiating ESCC from PMME. The radiomics signature-based nomogram model was a statistical instrument that provided a probability of PMME. The aim of this nomogram was to discriminate between patients with PMME and those with ESCC. This method provided the oncologists or radiologists with advice on whether an esophageal neoplasm was a PMME or an ESCC. When this radiomics nomogram indicated the diagnosis of PMME for an esophageal neoplasm in clinical practice, it required careful observation with endoscopy when appropriate. Further, a deeper esophageal biopsy and histological examination with immunohistochemical analysis of the biopsy specimen are recommended in order to acquire an appropriate diagnosis.

Among esophageal neoplasms, ESCC was only chosen to distinguish it from PMME, because the ESCC was the most common lesion among esophageal neoplasms and PMME may be misdiagnosed as ESCC sometimes in clinical practice. The other esophageal neoplasms included leiomyoma, GIST, lymphoma, metastasis, and neurofibroma [30]. The most prevalent of these neoplasms was esophageal leiomyoma. The radiologic features of leiomyomas were an expansive growth pattern, a smooth tumor surface, noninfiltrative to peritumoral fat space, the presence of calcification, slightly homogeneous enhancement without necrosis, and so on [10, 30]. The esophageal leiomyoma was easily differentiated from PMME using CT imaging in clinical practice. The other neoplasms, such as GIST, lymphoma, and metastasis, were extremely rare. So, we just chose ESCC for differentiating from PMME.

There were some limitations to the current investigation. First, due to the rarity of the PMME, the sample size was small; a significantly larger sample for multicenter external validation was required to demonstrate the benefit and repeatability of this radiomics nomogram model. Second, this research did not include additional imaging modalities, such as MRI. Third, this study did not incorporate radiomic signatures and CT findings to distinguish PMME from ESCC because the CT findings for diagnosing PMME were carried out in our other study [10]. Fourth, previous reports showed that PMME was nearly twice to three times as common in men as in women [6–8]. In our study, PMME had a nearly equal gender distribution, and this biased result in gender might be a result of the small sample size of PMME. Fifth, the survival analysis of PMME patients using radiomics was not evaluated for resectable PMME in this study because not all patients received surgery due to aggressive biological behavior and inadequate availability of patients for receiving surgery to establish radiomics model. Sixth, there were no effective and reliable biomarkers to improve the detection, diagnosis, and monitoring of the therapeutic response of esophageal neoplasms. The routine tumor biochemical factors were not performed for the patients with esophageal neoplasms in clinical practice, so we could not select laboratory biochemical factors to conduct the radiomics nomogram. Our next research will concentrate on determining the efficacy of radiomics in predicting clinical outcomes in resectable PMME by analyzing survival and recurrence risk.

## 5. Conclusion

In conclusion, the proposed radiomics nomogram model based on CT could be used for distinguishing PMME from ESCC. As a diagnostic model, the radiomics nomogram model may contribute to assisting clinicians in determining an appropriate therapeutic schedule for the patients of esophageal neoplasms.

## Figures and Tables

**Table 1:** Characteristics of patients in primary and validation cohorts.

Characteristics	Primary cohort (*n* = 81)	Validation cohort (*n* = 41)	*T* or *χ*^2^	*P* value
Age (years)	62.46 ± 8.434	62.80 ± 8.119	0.218	0.828
Gender, *n* (%)			0.113	0.737
Male	61 (75.3%)	32 (78.0%)		
Female	20 (24.7%)	9 (22.0%)		
Pathology, *n* (%)			0.035	0.852
ESCC	62 (76.5%)	32 (78.0%)		
PMME	19 (23.5%)	9 (22.0%)		
Location, *n* (%)			3.122	0.373
Neck	2 (2.5%)	2 (4.9%)		
Upper-thorax	9 (11.1%)	8 (19.5%)		
Mid-thorax	32 (39.5%)	11 (26.8%)		
Low-thorax	38 (46.9%)	20 (48.8%)		

ESCC: esophageal squamous cell carcinoma; *n*: number; PMME: primary malignant melanoma of the esophagus. Numbers of patients presented as *n* (%). Data in parentheses are percentage of patients.

**Table 2:** Parameters of radiomics analysis.

Feature name	Image	AUC
Original_shape_sphericity	Plain CT	0.668 (0.551-0.785)
Wavelet-HLH_glrlm_low gray level run emphasis	Plain CT	0.647 (0.536-0.759)
Wavelet-HLH_glszm_size zone nonuniformity normalized	Plain CT	0.685 (0.572-0.799)
Wavelet-HLL_glcm_maximum probability	Plain CT	0.705 (0.597-0.813)
Wavelet-LHH_firstorder_minimum	Plain CT	0.619 (0.497-0.740)
Wavelet-HLH_firstorder_maximum	Enhanced CT	0.639 (0.513-0.765)
Wavelet-HLL_ngtdm_busyness	Enhanced CT	0.707 (0.584-0.829)
Wavelet-LHH_glcm_autocorrelation	Enhanced CT	0.676 (0.558-0.793)
Wavelet-LHL_glszm_gray level nonuniformity normalized	Enhanced CT	0.676 (0.562-0.789)

CT: computed tomography; AUC: area under curve. Data in parentheses are 95% confidence intervals.

**Table 3:** The performance of radiomics nomogram model for discriminating PMME from ESCC.

	Primary cohort	Validation cohort
AUC	0.975 (0.948-1.000)	0.906 (0.770-1.000)
Accuracy	91.4% [74/81] (85.2-97.5%)	92.7% [38/41] (84.7-100.0%)
Sensitivity	94.7% [18/19] (74.0-99.9%)	88.9% [8/9] (51.8-99.7%)
Specificity	90.3% [56/62] (80.1-96.4%)	93.8% [30/32] (79.2-99.2%)
PPV	75.0% [18/24] (53.3-90.2%)	80.0% [8/10] (44.4-97.5%)
NPV	98.2% [56/57] (90.6-100.0%)	96.8% [30/31] (83.3-99.9%)

AUC: area under curve; ESCC: esophageal squamous cell carcinoma; NPV: negative predictive value; PMME: primary malignant melanoma of the esophagus; PPV: positive predictive value. Data in parentheses are 95% confidence intervals.

## Data Availability

The data used to support the findings of this study are included within the article.

## Supplementary Materials

Supplementary Materials The supplementary table S1 shows the comparison of the clinical characteristics between PMME and ESCC.
