# Supplementary material for: A CT-Based Radiomics Nomogram Model for Differentiating Primary Malignant Melanoma of the Esophagus from Esophageal Squamous Cell Carcinoma
Source: Biomed Res Int. 2023 Feb 20;2023:6057196. doi: 10.1155/2023/6057196 (PMC9970707; doi:10.1155/2023/6057196)
Supplement: Supplementary Materials — The supplementary table S1 shows the comparison of the clinical characteristics between PMME and ESCC. [file 6057196.f1.docx]

Table S1. Characteristics of patients with ESCC and PMME

| Characteristics | ESCC (n=94) | PMME (n=28) | T or χ^2^ | P value |
| --- | --- | --- | --- | --- |
| Age | 63.39±7.029 | 59.82±11.320 | 1.581 | 0.123 |
| Gender |  |  | 17.811 | 0.001 |
| Male | 80 (85.1%) | 13 (46.4%) |  |  |
| Female | 14 (14.9%) | 15 (53.6%) |  |  |
| Location, n (%) |  |  | 3.162 | 0.367 |
| Neck | 4 (4.3%) | 0 (0.0%) |  |  |
| Upper-thorax | 11 (11.7%) | 6 (21.4%) |  |  |
| Mid-thorax | 35 (37.2%) | 8 (28.6%) |  |  |
| Low-thorax | 44 (46.8%) | 14 (50.0%) |  |  |

ESCC, esophageal squamous cell carcinoma; n, number; PMME, primary malignant melanoma of the esophagus

Numbers of patients presented as n (%). Data in parentheses are percentage of patients.
